# Tissue-agnostic drug approvals: how does this apply to patients with breast cancer?

**DOI:** 10.1038/s41523-021-00328-3

**Published:** 2021-09-13

**Authors:** Luiza N. Weis, Sara M. Tolaney, Carlos H. Barrios, Romualdo Barroso-Sousa

**Affiliations:** 1 Instituto de Ensino e Pesquisa Hospital Sírio-Libanês, São Paulo-SP, Brazil; 2 Department of Medical Oncology, Dana-Farber Cancer Institute, Boston, MA USA; 3 Harvard Medical School, Boston, MA USA; 4 LACOG, Oncoclinicas, Porto Alegre, Brazil; 5 Oncology Center, Hospital Sírio-Libanês Brasília, Brasília-DF, Brazil

**Keywords:** Breast cancer, Predictive markers, Biomarkers

## Abstract

Precision medicine has provided new perspectives in oncology, yielding research on the use of targeted therapies across different tumor types, regardless of their site of origin, a concept known as tissue-agnostic indication. Since 2017, the Food and Drug Administration (FDA) has approved the use of three different agents for tumor-agnostic treatment: pembrolizumab (for patients with microsatellite instability or high tumor mutational burden) and larotrectinib and entrectinib (both for use in patients harboring tumors with *NTRK* fusions). Importantly, the genomic alterations targeted by these agents are uncommon or rare in breast cancer, and little information exists regarding their efficacy in advanced breast cancer. In this review, we discuss the prevalence of these targets in breast cancer, their detection methods, the clinical characteristics of patients whose tumors have these alterations, and available data regarding the efficacy of these agents in breast cancer.

## Introduction

Breast cancer (BC) represents the most frequently occurring cancer and is the leading cause of cancer-related deaths among female patients worldwide^1^. Although substantial progress in treatment has been made, metastatic breast cancer (MBC) remains an incurable disease, and the 5-year survival rate for stage IV breast cancer is approximately 25%^2^.

With the emergence of next-generation sequencing (NGS) tools, cancer genomic data have become more widely available, and cancer therapy has shifted from a purely histology-based approach toward incorporating a precision medicine-based approach. Novel oncogenic drivers and biomarkers have been described across different tumor types, leading to the development of targeted therapies and the design of innovative trials, many of which are evaluating tissue-agnostic therapies^3^.

**Fig. 1:**
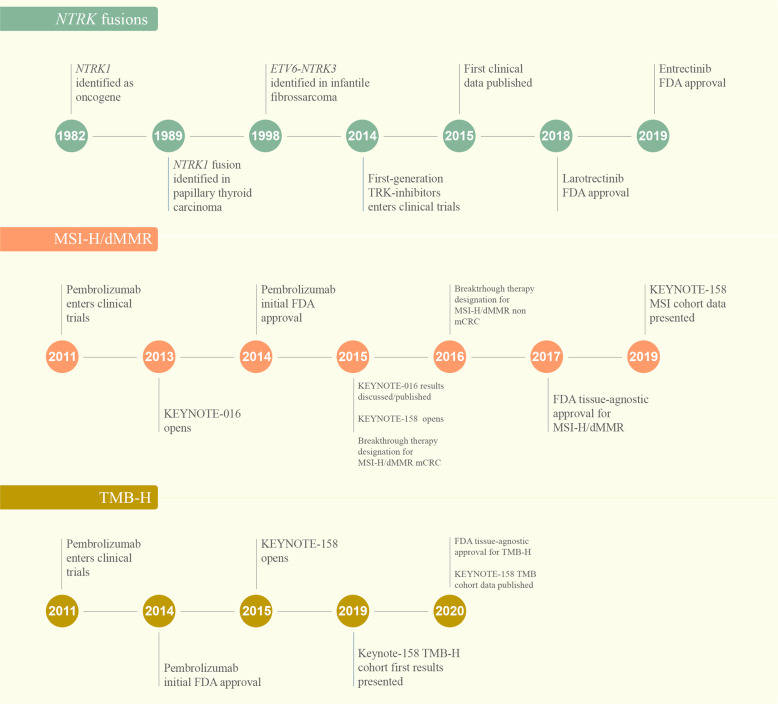
Timeline of discovery, validation, and clinical development of tissue-agnostic therapies. dMMR: mismatch repair deficiency, FDA: The United States Food and Drug Administration agency; MSI-H: microssatellite instability high, mCCR: metastatic colorectal cancer, TMB-H: tumor mutational burden high (cut-off ≥10 mutations/megabase).

The first tissue-agnostic treatment approval was granted by the FDA to pembrolizumab in patients with high microsatellite instability (MSI-H) tumors in 2017, followed by larotrectinib and entrectinib for the treatment of cancers harboring *NTRK* fusions in 2018 and 2019, respectively^4^. In 2020, the FDA expanded pembrolizumab approval to a new tissue-agnostic indication: high tumor mutational burden (TMB-H)^5^ (Fig. 1).

While these approvals were based on data from phase I and II trials conducted across several tumor histologies, only a few patients with MBC were included in those studies^6–8^. Therefore, we present an overview of the prevalence, detection methods, clinical characteristics, and data regarding the treatment efficacy of drugs targeting MSI-H/dMMR, *NTRK* fusions, and TMB-H in breast cancer. Finally, we also discuss the incorporation of these three biomarker tests and associated targeted therapies for MBC into routine clinical practice, including recommendations of medical societies and evidence of utility according to the ESMO Scale for Clinical Actionability of Molecular Targets (ESCAT) scale^9^.

## Mismatch repair deficiency (dMMR) and high microsatellite instability (MSI-H)

Microsatellites are repeated sequences of 1–6 nucleotides and are mostly located near the ends of chromosomes. They are particularly susceptible to acquired errors when the mismatch repair (MMR) system is impaired^10^. The MMR system represents one of the DNA repair pathways and corrects DNA base substitution mismatch, insertion or deletion, and slippage—conditions generated by DNA replication errors^11^. MMR deficiency (dMMR) arises due to mutations in at least one of the genes that encodes proteins in the MMR system (*MLH1, MSH2, MSH6*, and *PMS2*) or through methylation of the *MLH1* gene promoter that leads to MSI through accumulations of errors in DNA microsatellites^12^.

### How to determine MSI/MMR status

**Fig. 2:**
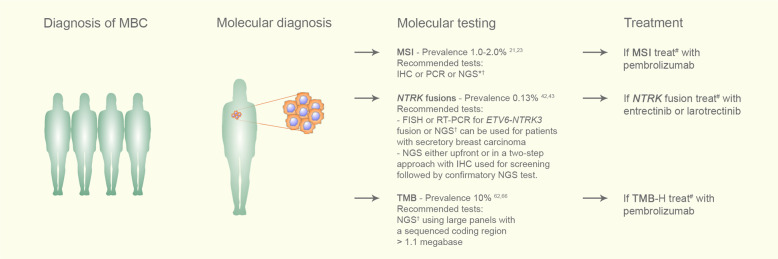
Suggested flowchart for testing and using approved tumor-agnostic therapies in patients with metastatic breast cancer. Following the diagnosis of metastatic breast cancer, formalin-fixed, paraffin-embedded (FFPE) tissue blocks from archival tissue or from a new tumor biopsy should be used to evaluate the status of one of the three discussed biomarkers: MSI, TMB-H, or NTRK fusions. In the case of TMB, blood samples can be collected to perform liquid biopsy-based NGS panels. For those patients harboring any of these three biomarkers who have progressed following prior treatment and who have no satisfactory alternative treatment options, we recommend the use of the appropriate tissue-agnostic approved therapy. IHC: immunohistochemistry; FFPE: formalin-fixed, paraffin-embedded; FISH: fluorescence in situ hybridization; MBC: metastatic breast cancer; NGS: next-generation sequencing; PCR: polymerase chain reaction. ^*^In cases of negative IHC for dMMR in breast cancer, confirmatory PCR or NGS could be performed. ^†^NGS should be performed preferentially at validated laboratories. #Treatment with the designated agnostic therapy should be started during the course of therapy for patients with the target biomarker who have progressed following prior treatment, and no satisfactory alternative treatment options are available.

Tumor MSI/MMR status can be tested using immunohistochemistry (IHC), polymerase chain reaction (PCR) and, more recently, NGS techniques (Fig. 2).

IHC for the MMR proteins MLH1, MSH2, MSH6, and PMS2 is a practical and widely available methodology among pathology laboratories. dMMR by IHC is defined by the absence of nuclear staining of some of the abovementioned MMR proteins in the tumor with preserved internal positive cell controls^10^. If IHC expression of at least one of those proteins is lost, the diagnosis of dMMR is established. Importantly, while there is a strong recommendation for performing IHC for all tumors belonging to the spectrum of Lynch syndrome, there are insufficient data for definitive recommendations in breast cancer. In cases of indeterminate IHC results, a molecular test preferentially based on PCR is indicated^13^.

The assessment of microsatellite alterations using classical PCR-based methodology combined with fragment length analysis is usually performed with DNA samples from tumors and paired normal DNA. This can be done using two panels: The National Cancer Institute (NCI) panel, which evaluates two single nucleotide repeat loci, BAT-25 and BAT-26, and three dinucleotide repeat loci, D2S123, D5S346 and D17S250; and pentaplex PCR, using five poly-A mononucleotide repeats (BAT-25, BAT-26, NR-21, NR-24, and NR-27). Tumors with instability at 2 or more of these markers are defined as MSI-H^10,12,14,15^. In fact, pentaplex PCR is the preferred panel given its higher sensitivity and specificity^13,16^. Frequently, clinical trials that assessed MSI/MMR status used both IHC and PCR methodologies.

More recently, the use of NGS panels has been validated for MSI-H diagnosis, and the reported concordance rates between NGS testing and IHC or PCR are both high^17,18^. While this can potentially become an alternative molecular test, with the advantage of simultaneous testing of TMB and other actionable gene mutations and optimization of tumor tissue usage, it is important to highlight that these tests should be performed at validated laboratories.

### Approval of pembrolizumab for treating MSI-H/dMMR cancers

Given that most MSI-H or dMMR tumors exhibit a hypermutated phenotype (>10 mutations per megabase) and that tumors with TMB-H present higher numbers of neoantigens that can potentially activate antitumor immunity, a hypothesis emerged suggesting that MSI-H tumors would be more suitable for immunotherapy.

In 2015, Le *et al*. published the results of KEYNOTE-016, a phase II trial designed to evaluate the clinical activity of pembrolizumab in 41 patients with treatment-refractory progressive metastatic carcinoma with or without dMMR. Pembrolizumab treatment resulted in increased objective response rates (ORRs) and superior progression-free survival (PFS) for dMMR colorectal and noncolorectal cancer patients compared to MMR-proficient colorectal cancer patients^19^. In an expanded phase of the abovementioned study, 86 patients with advanced dMMR metastatic solid tumors (across 12 different tumor types) who had been treated with at least one prior therapy received pembrolizumab. The primary endpoint, ORR, was 53%, and a complete response was achieved by 21% of patients^20^.

In May 2017, based on data from five studies, including the KEYNOTE-016, the FDA granted accelerated approval to pembrolizumab for the treatment of patients with advanced or metastatic MSI-H or dMMR colorectal cancer progressing on conventional therapy and other solid tumors also progressing after prior therapy without satisfactory alternative treatment options^5.12^.

More recently, data from the dMMR cohort of the KEYNOTE-158, a nonrandomized, open-label, multicohort, phase 2 study designed to evaluate pembrolizumab and predictive biomarkers in patients with advanced solid tumors, were presented. Overall, 233 patients with previously treated, advanced dMMR/MSI-H noncolorectal cancers who received pembrolizumab were included. After a median follow-up of 13.4 months, the ORR as the primary endpoint was 34.3%, the median PFS was 4.1 months, and the median OS was 23.5 months^6^.

### MSI-H/dMMR in breast cancer

Furthermore, little is known about the clinical features or prognostic value of MSI-H/dMMR in breast cancer as well as its association with response to ICIs (immune checkpoint inhibitors). Overall, the prevalence of MSI-H/dMMR in breast cancer is less than 2%^21–23^.

Fusco *et al*. evaluated the clinical value of MMR IHC and MSI analysis in 444 surgical breast cancer specimens, reporting 15% of dMMR tumors by IHC. When these tumors were analyzed by PCR with the five markers recommended by the National Cancer Institute, 91% were microsatellite-stable, suggesting that these methods might not be interchangeable in breast cancer^24^. Addressing the usefulness of MSI analysis in breast cancer, Siah and colleagues performed a tabulated survey that included 18,055 microsatellite analyses and found that greater than 300 different microsatellite markers were used to detect MSI in breast cancer. By restricting the survey to larger studies (>100 DNA samples analyzed), D11S988 was the most informative microsatellite marker, with a MSI-positive detection rate of 17.7%^25^. Such results stress the importance of performing studies to evaluate the concordance between different methods for identifying a tissue-agnostic biomarker.

A retrospective study in 228 Japanese patients with primary triple-negative breast cancer (TNBC) revealed a prevalence of 0.9% (two cases)^26^. Another retrospective study found four (1.8%) MSI-H/dMMR cases by IHC among 226 TNBC patients and none in 90 non-TNBC patients^27^. One tumor was classified as MSI-H/dMMR by PCR analysis, exhibiting hypermethylation of the MLH1 promoter. Notably, all four patients diagnosed with BC were over 50 years of age, had a family history of colorectal cancer, presented with high-grade tumors, and had invasive ductal carcinoma^27^. Horimoto and colleagues also evaluated MSI-H/dMMR in TNBC but focused on its relationship with tumor-infiltrating lymphocytes (TILs), especially in medullary carcinomas—a histological type enriched with TILs. Among 101 samples (63 classified as TIL-high TNBC and 38 as medullary carcinoma), there were no MSI-H/dMMR tumors^28^. Interestingly, unlike mucinous carcinoma of the colon, ovary, and endometrium, Lacroix-Triki *et al*. did not show an association between mucinous carcinoma of the breast and MSI^29^. Taken together, these data indicate that there are no clinical or pathological features in breast cancer that can be correlated with MSI.

Importantly, there is a paucity of data regarding the efficacy of pembrolizumab in patients with breast cancer harboring MSI-H/dMMR. Among 149 patients recruited from those five cohorts that provided data for pembrolizumab approval in MSI-H/dMMR metastatic cancers, only two had breast cancer, and both achieved partial responses, with durations of 7.6 and 15.9 months, respectively (Table 1)^5^. In KEYNOTE-158, five breast cancer patients were included in this trial, but their clinical characteristics and outcomes are unknown (Table 1)^5^. No patients with breast cancer were included in KEYNOTE-016^19,20^.

**Table 1:** Studies evaluating the efficacy of pembrolizumab, larotrecrinib and entrectinib among patients with breast cancer and MSI-H/dMMR, NRTK fusions or TMB-H alterations.

Molecular alteration	Reference	Study design	Treatment	Study population	Results
MSI-H/dMMR	KEYNOTE-016, 164, 012, 028, 158^5,6,19^	Pooled analysis of five phase II, open-label, single arm trials	Pembrolizumab 200 mg q3w or 10 mg/kg q2w	*N* = 149 CRC and non-CRC 2 BC pts	ORR 39.6% (general cohort) 2 PR in BC pts DOR (BC) range 7.6 to 15.9mo
	KEYNOTE-158^6^	Phase 2, non-randomized, open-label	Pembrolizumab 200 mg q3w	*N* = 233 5 BC pts	ORR 34% (general cohort), mDOR NR, mTTR 2.1mo Data from BC unknown
NTRK	LOXO-TRK-14001, NAVIGATE, SCOUT^40^	Expanded pooled analysis of three phase I/II trials in adults and pediatric pts	Larotrectinib 100 mg twice daily q28d (phase 2 dose)	*N* = 159 5 BC pts	ORR 79% (general cohort) ORR 75% BC
	Meric-Bernstam et al.^60^	Case series: NAVIGATE (1 pts) and compassionate use-protocols (5 pts)	Larotrectinib 100 mg twice daily q28d	*N* = 5 BC pts 2 TNBC pts 4 pts ETV6-NTRK3	ORR 80% (4/5 PR) All responses within 2 cycles
	ALKA-372-001, STARTRK-1, STARTRK-2^8,41^	Pooled analysis of three phase I/II trials	Entrectinib 600 mg once a day	*N* = 54 6 BC pts	ORR 57% (general cohort) ORR 83% (BC), 2 CR, mDOR 4.2 to 14.8mo
TMB-H	KEYNOTE-158^5^	Pre-planned retrospective analysis	Pembrolizumab 200 mg q3w	*N* = 102 (TMB-H) 0 BC pts TMB-H cut-off ≥10 mut/Mb (FoundationOne CDx)	ORR 29%, mDOR NR
	TAPUR^65^	Phase II basket study	Pembrolizumab	*N* = 28 MBC pts TMB-H cut-off ≥ 9 mut/Mb (FoundationOne CDx in 71% of specimens)	ORR = 21%, DC = 37% mPFS 10.6w, mOS 30.6w
	KEYNOTE-119^66^	Phase 3 trial (exploratory analysis for TMB)	Pembrolizumab vs chemotherapy	*N* = 601 mTNBC 2^d^/3^d^ lines TMB evaluable for 253 pts (42%) 10% TMB-H (cut-off≥10 mut/Mb by FoundationOne CDx)	Positive association among TMB and PFS (*p* = 0.014) and OS (*p* = 0.018) for pembrolizumab arm
	IMpassion-130^71^	Phase 3 trial (exploratory retrospective analysis for TMB)	Atezolizumab(A) + nab-paclitaxel(nP) vs placebo(PL) + nP	*N* = 902 TMB evaluable for 579 pts (FoundationOne CDx)	mTMB 4.39 mut/Mb PFS (highest quartile; 7.02 mut/Mb) HR 0.31 (95% IC:0.17,0.57) in PD-L1 + vs HR 0.84 (95%IC:0.48,1.47) in PD-L1- OS (all TMB quartiles) HR 0.71 (95% IC:0.52, 0.97) in PD-L1 +
	Barroso-Sousa et al.^70^	Prospective cohort	Anti-PD-1/L1 inhibitors alone (23%) or plus chemo (58%) or targeted therapy (19%)	*N* = 62 mTNBC 18% TMB-H (cut-off≥10mut/Mb) TMB assessed with WES (France Study 2016, MBCproject andTCGA-BRCA) and NGS (DFCI-ONCOPANEL, MSK-IMPACT and VICC)	ORR 58% TMB-H vs 30% TMB-L (*p* = 0.09) mPFS 12.5mo TMB-H vs 3.7mo TMB-L (*p* = 0.03) mOS 29.2mo TMB-H vs 14.2mo TMB-L (p = 0.06)

q3w: every 3 weeks, q2w: every 2 weeks, CRC: colorectal cancer, BC: breast cancer, pts: patients, ORR: overall response rate, PR: partial response, DOR: duration of response, NR: not reached, mTTR: median time to response, mo: months, q28d: every 28 days, TNBC: triple negative breast cancer, DC: disease control, MBC: metastatic breast cancer, mPFS: median progression free-survival, mOS: median overall survival, mTNBC: metastatic triple negative breast cancer, pembro: pembrolizumab, chemo: chemotherapy, m: median, TMB-L: low mutational tumor burden.

## Neurotrophic tropomyosin receptor kinase (*NTRK*) fusions

The *NTRK1*, *NTRK2*, and *NTRK3* genes encode the neurotrophin receptors TRKA, TRKB, and TRBC, respectively, which are predominantly transcribed in the nervous system in adult tissues. The TRK family plays an important role in nervous system development through regulation of cell proliferation, differentiation, apoptosis, and survival of neurons in both central and peripheral nervous systems^30^.

Fusions involving these genes are the most common mechanisms of oncogenic TRK activation and are found in both adult and pediatric tumors. *NTRK* fusions are enriched in rare cancer types, including infantile fibrosarcoma, congenital mesoblastic nephroma, secretory breast carcinoma, and mammary analog secretory carcinoma. Common tumors, such as lung, melanoma, and colorectal cancers, have low frequencies of these genomic alterations^31^.

### How to determine *NTRK* fusions status

There are different methods for identifying *NTRK* fusions: fluorescence in situ hybridization (FISH), RT-PCR, and RNA- or DNA-based NGS^32^. Notably, IHC may be used as a screening method, as we will discuss below (Fig. 2).

The use of FISH or RT-PCR is not recommended as a screening tool and should be reserved for cases where *NTRK* fusions are highly recurrent (*ETV6-NTRK3* fusion) as in the case of infantile fibrosarcoma or secretory breast carcinoma^33^. FISH can be performed with break-apart probes for the three *NTRK* genes, which requires either separate or multiplex assays, or through a break-apart probe for the *ETV6* gene in cases that are histologically suggestive of *ETV6-NTRK3* fusions. FISH is not able to identify the gene fusion partner, requires expertise, and is more expensive when a multiplex assay is used. RT-PCR provides direct evidence of a *NTRK* fusion and detects only known fusion partners and breakpoints^15,32^.

RNA- or DNA-based NGS methods are able to assess NTRK fusions with the advantage of providing other important molecular information, including the presence of other oncogenic drivers, tumor mutation burden, and monitoring of patients for the development of resistance mutations. RNA-based NGS has some advantages over DNA, since it is an approach that allows *de novo* detection of gene fusion transcripts that have not been previously described and increases the sensitivity of detection in low tumor purity samples^33^.

For tumors that rarely harbor *NTRK* fusions, front-line NGS-based approaches or a two-step approach with IHC followed by sequencing tests are indicated^32^. IHC examines the expression of TRK proteins but does not directly detect NTRK fusions^34,35^. It can be performed through different antibodies, such as antibodies directed against specific NTRK proteins or a pan-TRK antibody cocktail^32^. Tumors are considered positive if ≥ 1% of tumor cells exhibit positivity at any intensity above the background^36^. The staining pattern is variable in intensity and localization (nucleus, cytoplasm, or membrane), showing a correlation with the fusion partner^34^. IHC has shown high sensitivity (from 93%^34^ to 100%^37^) and specificity (from 93%^37^ to 100%^34^) for the detection of NTRK fusions. Consequently, IHC, when positive, may be used to enrich patients with NTRK fusions as part of a two-step detection process. Importantly, IHC shows lower sensitivity for NTRK3 fusions and lower specificity for tumors with neuronal and muscular differentiation^38^.

### Approval of larotrectinib and entrectinib for treating tumors with NTRK fusion

In November 2018, the FDA granted accelerated approval for larotrectinib—a potent and selective inhibitor of all three TRK proteins—for adult and pediatric patients with solid tumors harboring *NTRK* gene fusion without a known acquired resistance mutation with metastatic disease or for whom surgical resection would likely result in severe morbidity, and who had no satisfactory alternative treatments available or whose cancer had progressed following treatment^39^. This approval was based on the results of three multicenter, open-label, single-arm clinical trials that evaluated 55 patients treated with larotrectinib and demonstrated an ORR of 75%, with median duration of response and PFS not reached^7^. In an expanded pooled efficacy analysis of 159 patients recently published, the ORR was 79%, and complete responses were achieved in 16% of patients with a median PFS of 28.3 months and median OS of 44.4 months^40^.

In 2019, another *NTRK* inhibitor, entrectinib, was granted FDA approval for the same therapeutic indications as larotrectinib. Data from a pooled analysis from three phase 1-2 trials, including 54 patients with advanced or metastatic disease presenting with an *NTRK* fusion, showed an objective response of 57% for the overall population, with 7% of patients achieving a complete response with a median PFS of 11.2 months and a median OS of 21 months^8,41^.

### *NTRK* fusions in breast cancer

*NTRK* fusions represent a rare molecular alteration in breast cancer. Ross *et al*. identified only 16 tumors (0.13%) with *NTRK* gene fusions among 12,214 locally aggressive, relapsed, or metastatic BC using comprehensive genomic profiling. Among them, nine cases were ductal carcinomas and three were secretory carcinomas. All tumors were HER2 negative, more commonly TNBC, and the majority had *NTRK1* fusions^42^.

Conversely, human secretory breast carcinoma represents a rare subtype of invasive carcinoma (less than 0.02% of all breast cancers)^43^ described in pediatric and adult populations that very frequently (above 90%) harbor *ETV6-NTRK3* gene fusion^44^. Genomic profiling of secretory breast carcinoma showed that most cases are classified as basal-like tumors with triple-negative receptor status^45,46^. However, *ETV6*-*NTRK3* gene fusion is usually associated with indolent, slow-growing tumors with favorable prognosis, highlighting the molecular heterogeneity of triple-negative tumors^47,48^. A small series of 24 cases of secretory breast carcinoma evaluating pan-TRK IHC as a diagnostic assay found a positivity expression rate of 95.8%. A pattern of diffuse and/or at least focally strong nuclear staining was a sensitive (83.3%) and specific (100%) diagnostic marker for this entity and may provide a more rapid and cost-effective test than FISH or NGS^49^.

To date, there is information about the efficacy of these agents in 15 patients with MBC, with response rates of approximately 80% (Table 1)^8,40,50^. Tumors known to have a high frequency of *NTRK* fusions, such as secretory breast carcinoma, should always be evaluated for the presence of this molecular driver using RT-PCR or FISH as initial methods. For tumors with a low pretest probability of a positive result (e.g., breast cancers in general), a cancer gene panel would be a better test to exclude other more common targetable molecular alterations (Fig. 2). Treatment of MBC harboring *NTRK* fusions with TRK inhibitors is approved for patients with progressive disease despite previous treatment and conveys impressive response and survival rates in initial studies. Further research should address the role of TRKi in earlier lines of therapy, especially in *NTRK*-enriched histologies.

## Tumor mutational burden

TMB can be defined as the total number of somatic mutations per megabase (mut/Mb) of the genome examined^51,52^. Tumors with high TMB have a high neoantigen burden, some of which might increase T-cell reactivity^53^. Thus, it was hypothesized that tumors with high TMB are more responsive to immune checkpoint inhibitors (ICIs). In fact, several retrospective and prospective studies have suggested that high TMB is associated with improved response to ICIs in several tumor types^54–57^.

The predictive role of TMB in the benefit of ICIs has been surrounded by controversy. This can be partially explained by the fact that TMB has been calculated using different platforms. In addition, TMB is influenced by tumor purity, ploidy, sequencing depth of coverage, and analytic methodologies. Furthermore, the threshold definition of high TMB is still not optimized across cancer types^58^. Additionally, while the use of large panels (covering > 1.1 megabase of the sequenced coding region) has been validated in the context of clinical trials, it is important to highlight that their use tends to overestimate the mutational burden compared with whole exome sequencing. Multiple ongoing initiatives are attempting to standardize TMB assessment, and further work is necessary to establish the best cut-off for using TMB as a predictive biomarker of response to immunotherapy^51,53,59^.

### How to determine TMB in clinical practice

Different large NGS panels are approved/authorized by the FDA and provide TMB evaluation: (1) tissue-based FoundationOne CDx (F1CDx), which defines TMB as the number of base substitutions (including synonymous mutations) in the coding regions of targeted genes;^60^ (2) tissue-based MSK-IMPACT, which tabulates nonsynonymous mutations using data from both tumor and germline DNA;^55^ (3) liquid biopsy-based Guardant360; and (4) the liquid-based biopsy FoundationOne Liquid CDx^61^.

### Approval of Pembrolizumab for treating TMB-H cancers

In June 2020, the FDA expanded the approval of pembrolizumab to include unresectable or metastatic tumors with TMB-H (≥10 mut/Mb) that have progressed following prior treatment and that have no satisfactory alternative therapy options. The FDA also approved the FoundationOneCDx assay as a companion diagnostic for pembrolizumab^5^. The approval was based on a preplanned retrospective analysis of KEYNOTE-158. Among 790 patients who received pembrolizumab and had sufficient tissue for TMB analysis, 102 (13%) were identified as having TMB-H tumors. The ORR was 29% for TMB ≥ 10 mut/Mb compared to 6% for the non-TMB-H group and was maintained even when MSI-H tumors were excluded. The median duration of response was not reached^5^.

### TMB in breast cancer

Using publicly available genomic data from 3969 patients with breast cancer, Barroso-Sousa *et al*. showed that while the median TMB of these tumors was 2.63 mut/Mb, 5% of breast cancers had high TMB (cut-off of ≥ 10 mut/Mb), and metastatic tumors had a greater prevalence of high TMB than primary tumors (8.4% vs. 2.9%). TNBC had a significantly higher median TMB than hormone receptor-positive or HER-2-positive cancer, although the frequency of tumors with high TMB did not differ between subtypes. In addition, it was shown that high TMB tumors had higher RNA expression of the CD8-positive T-cell effectors GZMA and PRF1 with greater immune cytolytic activity, suggesting that these patients are more likely to respond to ICI therapies^62^. Interestingly, enrichment of hypermutation was significantly higher in metastatic invasive lobular carcinoma than in invasive ductal metastatic tumors, a finding also described by Sokol and colleagues^62,63^. Importantly, other studies confirmed that the prevalence of TMB-H in breast cancer is approximately 10%, which is far from being a rare alteration^64^. Although the TMB-H threshold is still debated, the cut-off of ≥10 mut/Mb was the most used in studies evaluating TMB in breast cancer^62,65–67^.

Given that MMR defects represent an important mechanism leading to hypermutation, it would be of interest to address whether there is an overlap between high TMB and MSI status in breast cancer. However, there is a paucity of data on this issue. Similar to Chumsri^67^ et al., our group previously showed that the APOBEC signature is the most common dominant mutational process associated with hypermutation in breast cancer, followed by the dMMR signatures^62^. The APOBEC family has enzymatic activity that is essential for innate and adaptive immune responses, and its upregulation or mutation can be used as a potential predictive marker for immunotherapy responses in non-small cell lung cancer^68^.

With respect to the role of TMB as a biomarker, one study demonstrated that TMB-H was an independent prognostic factor, with longer OS observed in HER2-positive MBC previously treated with standard treatment^69^. In mTNBC patients treated with anti-PD-1/L1 therapies, Barroso-Sousa and colleagues observed a significant positive association of TMB-H with longer progression-free survival (PFS) independent of clinical factors and PD-L1 status, and there was a trend toward improved OS^70^.

To date, the only study designed to prospectively evaluate the use of ICIs in patients with MBC and high TMB was the phase II basket study TAPUR. In this study, a cohort of 28 patients with MBC and TMB of ≥ 9 received pembrolizumab. Of note, 93% of patients were treated with ≥3 prior systemic therapies, and 46% were TNBC (Table 1). TMB-H ranged from 9 to 37 mut/Mb, disease control was obtained in 37% of patients, the ORR was 21%, and the median PFS and OS were 10.6 and 30.6 weeks, respectively^65^.

An exploratory analysis from KEYNOTE-119—a randomized, open-label, phase III study of pembrolizumab monotherapy versus single-agent chemotherapy in previously treated mTNBC patients—revealed a prevalence of TMB-H, defined as ≥10 mut/Mb, of 10% in the evaluable population. A positive association was established among TMB and PFS and OS for patients treated with pembrolizumab but not for those who underwent chemotherapy^66^.

Data on TMB were also analyzed in patients included in the randomized, placebo-controlled, phase 3 trial Impassion130, which evaluated the combination of atezolizumab plus nab-paclitaxel or placebo plus nab-paclitaxel in patients with untreated mTNBC^71^. There was no correlation between TMB and PD-L1 status, and the prevalence of patients with TMB-H was 8%. In this study, higher TMB levels were associated with improved PFS and OS in the atezolizumab versus placebo arm in patients with PD-L1 + tumors but not in those with PD-L1– tumors. There was no information about the benefit specifically in the TMB-H (≥10 mut/Mb) population.

Regardless of ICIs (combined with chemotherapy) being approved for patients with PD-L1-positive mTNBC, it would be of clinical interest to investigate 1) whether patients with other breast cancer subtypes and TMB-H, such as estrogen receptor-positive tumors, could also benefit from ICIs and 2) whether patients with TMB-H PD-L1-positive mTNBC could be treated solely with immunotherapy and be spared from chemotherapy. NIMBUS (NCT03789110), an ongoing phase 2 trial, is recruiting patients with HER2-negative MBC whose tumors have a TMB ≥ 9 mut/Mb for treatment with a combination of nivolumab and ipilimumab^72^.

## Translating these data into clinical practice

To aid oncologists in defining the utility of target treatments according to specific molecular alterations, ESMO developed the ESCAT scale, classifying molecular drivers into six tiers (I-V and X) based on the level of clinical evidence and providing guidance for treatment with targeted therapies^9^. In 2019, Condorelli *et* al. classified molecular drives in BC according to this scale: MSI and NTRK were assigned to tier IC. In this category, a target is suitable for routine use, and the strength of evidence is based on clinical trials conducted in multiple tumor types or basket trials that demonstrated clinical benefit for the target-drug pair with a similar magnitude of benefit across different tumor types^73^. With regard to TMB, there is no formal ESCAT designation from the breast cancer panel.

While searching for these particular molecular alterations in MBC, it is important to consider aspects including the prevalence of the molecular driver, accuracy, cost, and availability of different detection methods and reimbursement or regional regulatory policies regarding access to treatment. Although NGS panels are increasingly being used and able to identify multiple drivers at the same time, their costs are still not affordable for many patients or not covered by some payers. Additionally, patients must be aware that the chance of finding a druggable alteration is low. At the present time, some guidelines do not recommend NGS for advanced breast cancer in routine clinical practice^74,75^. On the other hand, it is important to highlight that FDA tissue-agnostic approvals have focused on patients who have progressed following prior lines of treatment and have no satisfactory alternative therapeutic options in the metastatic setting. Taking all these pros and cons into account, routinely performing tests for evaluating the status of these three biomarkers for patients with MBC is recommended if feasible (Fig. 2).

## Future perspectives

It is becoming clear that for any target, there is a spectrum of actionability that extends from histology-specific to histology-agnostic biomarkers and that this degree of actionability has a dynamic nature that will need continuous revision with the emergence of new drugs and regimens^76^. This highlights the need for additional data on the efficacy of drugs targeting specific biomarkers in patients with advanced breast cancer.

Given their low frequency in breast cancer, a feasible alternative to obtaining more data on the effectiveness of agents targeting MSI-H/dMMR and *NTRK* fusions could be to obtain real-world data. An international registry capturing experience from physicians around the world would be of value in this situation. With regards to TMB-H, the scenario is different. The prevalence of TMB-H in MBC is approximately 10% (far from being considered a rare genomic event), and it would be desirable to generate prospective, randomized data on the effectiveness of immunotherapy in MBC patients, similar to what has been done in other rare molecular drivers such as ALK rearrangements^77^ or RET fusion^78^ in nonsmall cell lung cancer.

In addition, other molecular alterations have potential for histology-agnostic designation, including RET alterations, BRAF mutations, fibroblast growth factor receptor (FGRF) aberrations, KRAS 12 G, ROS1, ALK, NRG1, HER2, and POLE/POLD1^76,79,80^.

## Conclusion

The emergence of tissue-agnostic therapy in oncology is the apex of precision oncology. Since 2017, the FDA has approved the use of three different agents for tumor-agnostic treatment: pembrolizumab for patients with microsatellite instability or high tumor mutational burden, larotrectinib, and entrectinib—both for use in patients harboring tumors with *NTRK* fusions. However, only a few patients with MBC were included in the pivotal trials that led to FDA approval of these agents. Despite the limitations regarding the low representation of patients with MBC in pivotal studies, we recommend routinely performing tests to evaluate the status of these three biomarkers among patients with MBC, if feasible. However, given that the spectrum of actionability of these drivers can vary according to the tumor type, additional data assessing the degree of benefit and optimal treatment sequence in patients with MBC harboring these genomic alterations are needed.
